# Chemistries and applications of DNA-natural product conjugate

**DOI:** 10.3389/fchem.2022.984916

**Published:** 2022-09-06

**Authors:** Yuanyuan Chen, Wenting Li, Hang Xing

**Affiliations:** Institute of Chemical Biology and Nanomedicine, State Key Laboratory of Chemo/Biosensing and Chemometrics, College of Chemistry and Chemical Engineering, Hunan University, Changsha, Hunan, China

**Keywords:** natural product, functional DNA, DNA-drug conjugate, aptamer, targeted therapy

## Abstract

Natural products and their derivatives have made great contributions to chemotherapy, especially for the treatment of tumors and infections. Despite the achievements, natural product-based small molecule drugs usually suffer from side effects, short circulation time, and solubility issue. To overcome these drawbacks, a common approach is to integrate another bio-functional motif into a natural product compound, enabling targeted or synergistic therapy. One of the most promising strategies is to form a DNA-natural product conjugate to improve therapeutic purposes. The incorporated DNA molecules can serve as an aptamer, a nucleic-acid-based congener of antibody, to specifically bind to the disease target of interest, or function as a gene therapy agent, such as immuno-adjuvant or antisense, to enable synergistic chemo-gene therapy. DNA-natural product conjugate can also be incorporated into other DNA nanostructures to improve the administration and delivery of drugs. This minireview aims to provide the chemistry community with a brief overview on this emerging topic of DNA-natural product conjugates for advanced therapeutics. The basic concepts to use the conjugation, the commonly used robust conjugation chemistries, as well as applications in targeted therapy and synergistic therapy of using DNA-natural product conjugates, are highlighted in this minireview. Future perspectives and challenges of this field are also discussed in the discussion and perspective section.

## Introduction

The development of more effective drugs is the central topic in chemistry and biotechnology. In addition to modern drug discovery methods in the pharmaceutical industry, such as high throughput screening assays (David et al., 2014), DNA-encoded libraries (Huang et al., 2022; Sunkari et al., 2022), and artificial intelligence (AI)-assisted machine-learning technology (Bowen et al., 2020; Feng et al., 2022), high potent natural products isolated from plants and other natural sources remain an important source of new chemical entity drugs (Frantz and Smith, 2003). To date, natural products and natural products derivatives comprise 26% of new drugs being developed (Newman and Cragg, 2020). These natural product drugs have demonstrated their key role against many long-established druggable targets including kinases, proteases, ion channels, G protein-coupled receptors, and other functional enzymes, making remarkable contributions to the treatment of cancer (Okouneva et al., 2003), acquired immunodeficiency syndrome (AIDS) (Yu et al., 2003), Alzheimer’s disease (Heinrich and Lee Teoh, 2004) and malaria (Cowman et al., 2016). Despite these achievements, the drawbacks of natural product drugs are non-negligible. One of the main downsides of natural products is off-target which leads to severe side effects and undesired toxicity. Some natural products also have a short half-life span and can be rapidly eliminated by systemic circulation in the biliary and renal systems (Ahuja et al., 2020; Zhang et al., 2020). This decreases the bioavailability of natural product drugs, and thus repeated administration is necessary (Butler et al., 2014). Another disadvantage is the poor solubility of natural medicines. For example, natural paclitaxel shows limited effectiveness in aqueous media due to its inherent insolubility. One approach to improve its solubility and effectiveness is to covalently link the drug onto gold nanoparticles via a DNA linker. The resulting conjugates are highly soluble in aqueous buffers, showing more than 50-fold higher solubility than unconjugated drug (Zhang et al., 2011; Ahuja et al., 2020; Jain and Chella, 2020).

To alleviate the drawbacks of natural products, a variety of conjugation technologies integrating the natural product with another functional motif have been developed, including small-molecule drug conjugate (Krall et al., 2014; Zhuang et al., 2019), peptide drug conjugate (Ma et al., 2017; Wang et al., 2017), protein-drug conjugate (Vhora et al., 2015), antibody-drug conjugate (ADC) (Strebhardt and Ullrich, 2008; Beck et al., 2017; Bardia et al., 2019; Drago et al., 2021), virus-like drug conjugate, etc. For example, monomethyl auristatin E (MMAE) is a dolastatin-10 pentapeptide derivative with microtubule-disrupting ability. Because of its high toxicity, it cannot be used as a drug itself; instead, it has been linked to an anti-CD30 antibody to target CD30-positive cancer cells (Fanale, 2017; Chen et al., 2020). This ADC formulation, with the commercial name of Brentuximab Vedotin, has been approved by FDA in 2018 for the treatment of various lymphomas and mycosis fungoides. The “ligand-linker-drug” construct has been demonstrated as a highly effective formulation to enhance the location-specificity and half-life span of natural products, reducing toxicity in healthy tissues and increasing efficacy.

In addition to the above-mentioned technologies, owing to the tremendous achievements of DNA technology over the past decades, conjugating DNA into natural products emerges as one of the most promising strategies to improve therapeutic purposes. Pioneered by Seeman, Mirkin, et al., DNA has been recognized as a programmable material to precisely assemble cargo molecules in 3D space with a single base pair spatial control (Jones et al., 2015). The organization of natural product molecules into a highly ordered DNA nanostructure can largely improve the efficiency of drug delivery. More importantly, the discovery of functional DNA, including aptamer and DNAzyme, provides diverse “chemical analogues” to native antibodies and enzymes with similar recognition and catalytic properties (Liu et al., 2009; Jia et al., 2022). Functional DNA is also considered to be of high stability, low immunogenicity, and easy chemical modification (Liang et al., 2014; Li et al., 2016). Thus, the integration of natural products and functional DNA has demonstrated the potential to realize many advanced therapeutic approaches, including targeted cancer therapy, synergistic chemo-immunotherapy, and targeting previous “undruggable” biotargets (Zhu et al., 2015; Chen et al., 2017; Kim et al., 2021). In this minireview article, we focus on the topic of DNA-natural product conjugate and aim to provide a brief overview of the synthetic chemistries and biomedical applications of this emerging conjugate, especially the use of it in the field of cancer therapy. At last, we provide our envisions on the future development of DNA-natural product conjugate.

## DNA–Natural product conjugation chemistry

**FIGURE 1:**
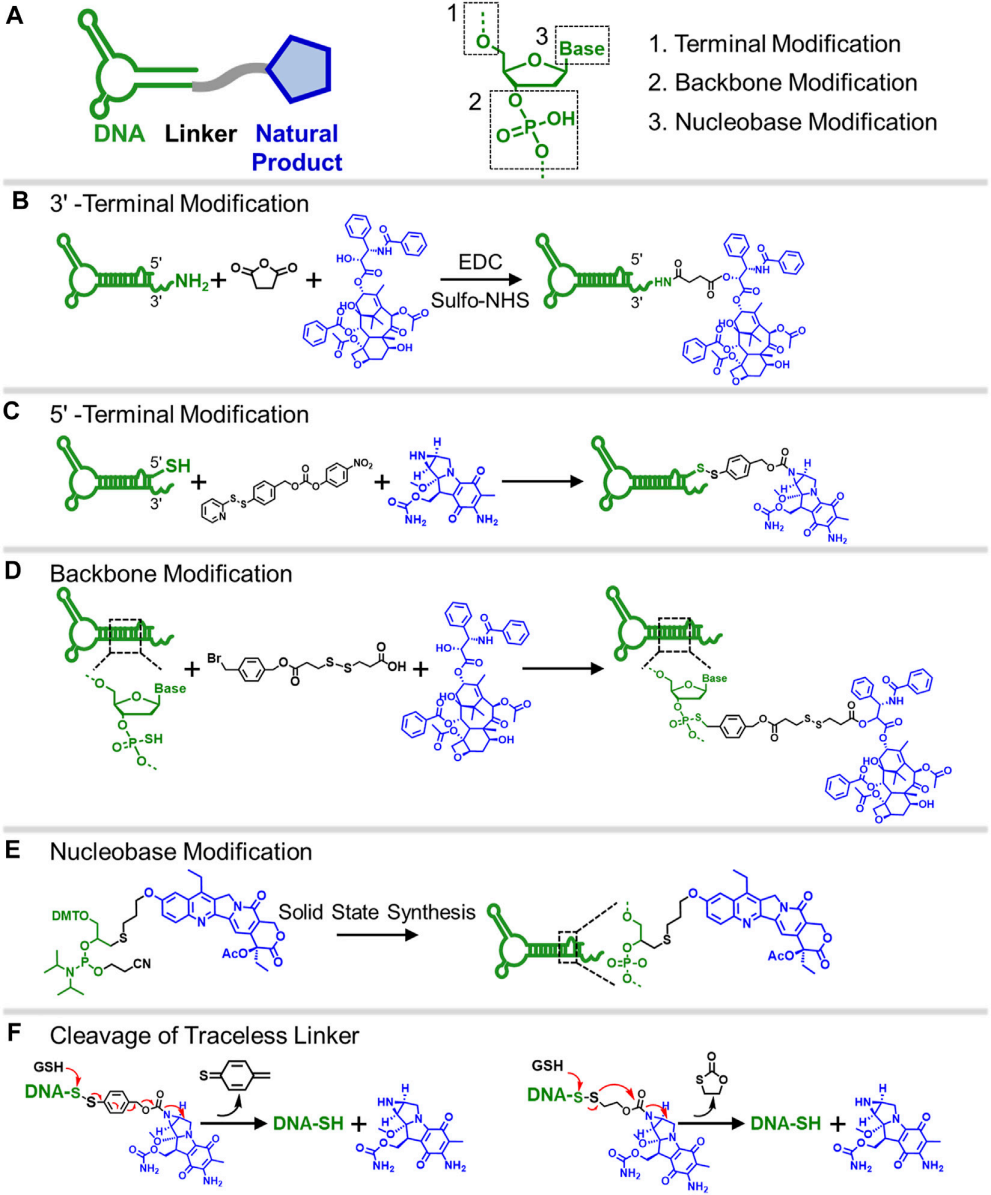
**(A)** Scheme showing the “ligand-linker-drug” construct and three commonly used modification sites on a DNA nucleotide for conjugation. **(B)** Conjugation of PTX on 3′-terminus of an amine-modified DNA. **(C)** Conjugation of MMC on 5′-terminus of a thiolated DNA. **(D)** Conjugation of PTX on phosphorothioate backbone using a hetero-bifunctional linker. **(E)** Use CPT-containing phosphoramidite to prepare a drug-loaded aptamer strand using solid-state synthesis. **(F)** Two commonly used traceless linkers to enable the release of native natural product drugs in the presence of GSH.

A DNA molecule is a biopolymer with a chain of nucleotide monomers consisting of a phosphate backbone, deoxyribose, and nitrogenous base group. A typical synthetic strategy to form the DNA-natural product conjugate is to use native functional groups on a natural product, such as ɑ-hydroxyl, phenol hydroxyl, and a primary amine, to covalently attach to DNA via homo- and hetero-bifunctional crosslinkers (Figure 1A). Based on the molecular structure of DNA, Figure 1 illustrates three commonly used modification chemistries to install natural product molecules onto DNA: 1) terminal modification (Figures 1B,C); 2) modification of phosphodiester backbone (Figure 1D); and 3) modification on nucleobase site (Figure 1E).

The first and most used conjugation chemistry is to immobilize natural products onto the 3′- or 5′-terminus of a DNA strand. Typically, the terminus of a DNA strand is modified with active thiol or primary amine to conjugate with natural product using a bifunctional crosslinker. Mirkin and colleagues developed a DNA-paclitaxel (PTX) conjugate to improve the drug solubility and efficacy (Zhang et al., 2011). The PTX was first reacted with succinic anhydride to install a carboxyl group on its active ɑ-hydroxyl. The DNA strand was synthesized with a primary amine on the 3′-terminus to react with carboxylated PTX using EDC/sulfo-NHS coupling chemistry (Figure 1B). The conjugate can be further immobilized onto gold nanoparticles, resulting in a nanoparticle drug delivery system that delivers drugs to cancer cells. Similarly, natural products can also be attached to the 5′-terminus of the DNA strand. Tan and colleagues used a hetero-crosslinking agent to conjugate the mitomycin C (MMC) with a 5′-tholated aptamer (Yang et al., 2020). The conjugation reaction occurs in the active secondary amine group on MMC, which does not affect the bioactivity of the drug (Figure 1C). They prepared three different aptamer-MMC conjugates and demonstrated these conjugates showed enhanced cytotoxicity against target cancer cells. The terminal modification approach is straightforward and effective, except that the number of attached drugs is limited. Approaches that can achieve a higher drug-to-DNA ratio are needed.

The second conjugation chemistry is to incorporate phosphorothioate (PS) at the desired position on the backbone of a DNA strand and attach the natural product to the PS sites. Since PS is chemically different from native phosphodiester (PO), site-selective drug modification can be realized. Zhang and colleagues used this approach to graft PTX molecules on the PS backbone of a DNA strand (Guo et al., 2019). The PTX was first modified with a benzyl bromide group and was then selectively attached to PS sites at the benzyl bromide-modified ɑ-hydroxyl site (Figure 1D). By using DNA sequence with PO and PS backbone blocks, amphipathic DNA-PTX conjugate with aptamer and PTX-PTX segments can be synthesized and further assembled into the spherical nucleic acid construct, enabling active tumor-specific delivery and drug resistance reversal for antitumor therapy. Zhang and colleagues further applied the PS backbone modification chemistry to prepare DNA-Camptothecin (CPT) conjugate to improve CPT aqueous solubility and delivery (Zhang J. et al., 2019). They used a disulfide linker to incorporate carbonethyl bromide to CPT on its -OH position, which can be covalently attached to the PS sites of DNA. The DNA-CPT strands were further assembled into a drug-loaded DNA tetrahedral structure with stimuli-responsive drug releasing properties.

The last method to prepare DNA-natural product conjugate is to use the drug-containing phosphoramidites to directly synthesize DNA strands with drug-modified nucleosides using standard solid-state synthesis. For example, Tan and colleagues prepared the CPT-containing phosphoramidites to synthesize a series of aptamer-CPT conjugates (Figure 1E) (Gao et al., 2021). The use of solid-phase synthesis can largely decrease the synthesis time and improve efficiency. They demonstrated that the aptamer sequence on the conjugate maintained targeting property and the incorporated CPT remained biological inhibitory activity. Cell studies revealed that aptamer-CPT conjugates can be specifically delivered to targeted HCT116 cells and released drugs intracellularly. The advantage of nucleobase modification and PS backbone modification is the ability to control the number and position of drug molecules on a DNA strand, thus enabling the possibility to develop precision medication for cancer and other diseases.

Covalent conjugation is stable and thus can reduce drug leakage. However, covalent modification might interfere with the binding ability of natural products, thus affecting their biological properties (Huang X. et al., 2021). One possible solution is to use non-covalent chemistry to prepare the DNA-drug conjugate that can be easily released in the desired location. One of the most used methods is the intercalation of drug molecules into double-stranded DNA (dsDNA). For example, doxorubicin (DOX), a common chemotherapy drug, can interact with dsDNA by occupation between planar base pairs using a tetracene ring (Stuart et al., 2014). By using aptamer as the targeting agent, DOX-intercalated dsDNA can be specifically delivered to the targeted cancer cell, showing enhanced antitumor efficacy in the mouse model (Zhu et al., 2013). Another approach is to use cleavable or traceless cleavable linker chemistry, which enables the release of the natural product when it reaches the target cell (Bräse and Dahmen, 2000). The stimuli-responsive drug release strategies include the reduction of disulfide bonds by glutathione (GSH), light-induced cleavage, and enzyme-catalyzed cleavage (Beck et al., 2017). Figure 1F illustrates two commonly used, disulfide-based, traceless cleavage linkers, 4-nitrophenyl 4-(2-pyridyldithio) benzyl carbonate (NPDBC) and 4-nitrophenyl 2-(2-pyridyldithio) ethyl carbonate (NPDEC). Under the condition of high intracellular GSH concentration, the broken disulfide bonds lead to the cleavage of the amide bond, releasing the native MMC molecule from the aptamer strand with the secondary amine group intact (Yang et al., 2020). The use of a traceless linker to prepare conjugate not only prevents acid-mediated degradation of MMC but also enables tumor microenvironment-responsive drug release. In addition to the above-mentioned traceless release strategies, other types of traceless linkers, such as amide-containing linker (Shannon et al., 2003; Gil and Brase, 2004), sulfone‐based chemistry (Cheng et al., 1999; Cheng et al., 2001; Huang T. et al., 2021), and aryl-triazene (Hejesen et al., 2013), can also be easily applied to synthesize DNA-drug conjugates, enabling targeted release of native natural products.

## Applications of DNA–Natural product conjugate

To date, DNA–natural product conjugate has been used for advanced therapeutic applications, showing enhanced efficacy over native drug molecules. One application is to construct DNA nanostructures, such as spherical nucleic acid, using DNA–natural product conjugate, improving the solubility, endocytosis ability, and bioavailability of the drugs. For example, Mirkin and colleagues developed polymeric SNA construct loading DOX via EDC/sulfo-NHS coupling chemistry (Banga et al., 2017). The SNA structure promotes cellular uptake of drugs, showing enhanced cytotoxicity against SKOV3 cancer cells. Another example was reported by Zhang and colleagues, who prepared PTX-loaded SNA-like micellar nanoparticles using amphiphilic blocked DNA-drug conjugates (Guo et al., 2019). Since multiple drug molecules can be attached to the phosphorothioate group on the DNA backbone, the PTX-SNA achieved a high drug loading ratio up to ca. 53%. The approach to assemble DNA-natural product conjugate into DNA nanostructure not only increases the stability and loading of the drug molecule but also improves its cellular uptake and half-life span, overcoming the key downsides of the small-molecule drug.

Another widely reported application is to use aptamer–natural product drug (ApDC) conjugate for targeted cancer therapy. Since aptamer serves as the antibody analogue that can bind target with high affinity and specificity, ApDC can help deliver drug specifically to the targeted sites, decreasing the side effects of chemotherapy. One of the first reported ApDC examples is the sgc8c-tethered DNA dsDNA loading with DOX. Theranostics (Zhu et al., 2013). The use of sgc8c aptamer enabled targeted delivery of DOX to protein tyrosine kinase 7 (PTK-7) positive CEM cells in mouse model, thereby achieving improved efficacy than DOX itself. Nucleolin is another common biomarker overexpressed on the surface of various cancer cells, which can be used as the potential binding target of aptamer (Soundararajan et al., 2009). Zhang and colleagues reported a nucleolin aptamer AS1411-PTX conjugation via a cathepsin B sensitive dipeptide bond. The conjugation can specifically target cancer cells to release PTX, showing antitumor ability, but did not affect the normal cell. Compared with non-targeted PTX, the AS1411-PTX conjugate presented higher cancer cell uptake and preferable targeting property in ovarian tumor mouse model (Li et al., 2017). Similarly, Tan and colleagues reported an AS1411-triptolide conjugate by coupling *p*-nitrophenylformate on triptolide and terminal amine on DNA. They demonstrated that triptolide can be specifically delivered and released into tumor cells, leading to lysosomal disintegration, and in turn, lysosomal-mediated programmed cell death (He et al., 2020).

In addition to the above-mentioned two applications, there is a growing interest to develop synergistic therapies by combining natural product chemotherapy with other treatments to overcome drug resistance. For example, P-glycoprotein or multidrug resistance protein (MDR1) overexpressed on cancer cells is one of the key issues affecting drug accumulation. To address this issue, Zhang and colleagues conjugated PTX onto a floxuridine (FdU)-integrated antisense oligonucleotide to develop synergistic chemo-gene therapy (Zhu et al., 2020). They used fluorescent dithiomaleimde as a linker to form the conjugation, which further assembled into an SNA construct. The conjugate can inhibit P-glycoprotein expression as well as release FdU and PTX, enabling synergistic therapy for drug-resistant cancer. Another promising approach for cancer treatment way is synergistic chemo-immunotherapy. Lv and colleagues reported the use of a DOX and CpG-loaded nanoparticle to modulate the tumor microenvironment for efficient chemo-immunotherapy (Dong et al., 2020). Compared with a hydrogel containing DOX only, the incorporation of CpG enabled a long-lasting immunostimulatory effect. The co-stimulation from both DOX and adjuvant can regulate the tumor microenvironment to a suppressive state, resulting in a stronger immune response and more effective chemo-immunotherapy. As we can see from these examples, the DNA-natural product conjugates largely expand the application regime of natural medicines in cancer treatment and other diseases.

## Perspectives

As discussed in this minireview and summarized in Table 1, conjugating natural product drugs to functional DNA represents one of the most promising methods to improve the therapeutic effects. Despite the growing interests and publications in this field over the past decade, the number of successful examples of DNA-drug conjugate in the pharmaceutical industry does not seem to correlate with its academic popularity, especially when compared with antibody-drug conjugate, which has already achieved tremendous success in clinic. To meet the challenges in bench-to-bedside transition of DNA-drug conjugate, more efforts shall be needed to address the critical issues affecting the therapeutic performance of DNA-based conjugates and the cost-efficacy benefit in the development.

**TABLE 1:** Representative examples of DNA–natural product conjugates.

Modification Method	Natural Product	Conjugate	Conjugation Chemistry	DNA Sequence	Disease	Ref.
Terminal Modification	Paclitaxel (PTX)	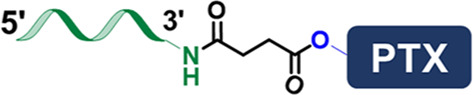	Coupling 3’-terminal amine and active hydroxyl on PTX using EDC/sulfo-NHS	DNA with oligo-T	Breast cancer	Zhang et al., (2011)
	Mitomycin C (MMC)	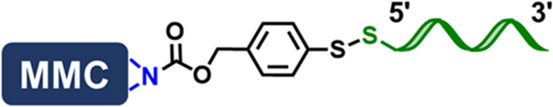	Coupling 3’-terminal thiol and secondary amine on MMC via hetero-bifunctional linker	XQ-2d	Prostate cancer	Yang et al., (2020)
Backbone Modification	Paclitaxel (PTX)	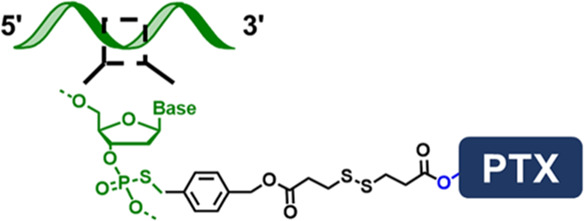	Modifying phosphorothioate group on DNA backbone using benzyl bromide linker	AS1411	Breast cancer	Guo et al., (2019)
	Camptothecin (CPT)	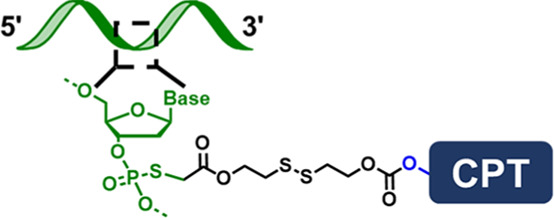	Modifying phosphorothioate group on DNA backbone using benzyl bromide linker	DNA tetrahedron	Colon cancer	Zhang J. et al., (2019)
Nucleobase Modification	Camptothecin (CPT)	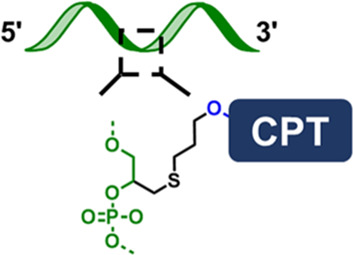	Using CPT-containing phosphoramidite to prepare a drug-loaded aptamer via solid-state synthesis	Sgc8-c	Colon cancer	Gao et al., (2021)
Non-covalent	Doxorubicin (DOX)	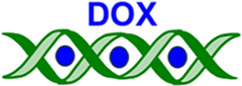	Intercalation between planar base pairs	Sgc8-c	Leukemia	Zhu et al., (2013)

First, the community might consider standardizing the evaluation procedures for DNA–natural product conjugates in animal models. Granted, the targeting effects and unique biological properties of functional DNA are robust and reproducible at the cellular level, but some of these properties might have interfered when reaching the *in-vivo* context (Sun et al., 2014; Toh et al., 2015). The formation of the protein corona, degradation of biomacromolecules, and inherent immune response may inevitably lead to the decrease of biorecognition properties. Thus, it is important to adapt appropriate controls and standardize the way conjugates are tested. For example, the scramble DNA sequence shall be an important control to verify the corresponding aptamer still binds tightly and specifically to its molecular targets in animal models (Xing et al., 2013). In this vein, the community can take advantage of the solid results and data of these conjugates to address potential issues associated with their use in therapy.

Second, more efforts are expected to address some key issues of functional DNA in therapeutic applications. Two widely-recognized issues are the relatively lower binding affinity (ca. 10^−6^ ∼ 10^−8^ M) and poorer pharmacokinetics and pharmacodynamics of DNA when compared with antibodies (Yan and Levy, 2018). Since different formulations might work differently in animal models, it might be important to test and analyze the performance of each DNA and its conjugate *in vivo* (Sun et al., 2014; Zhu and Chen, 2018; Zhang Y. et al., 2019; Odeh et al., 2019). A comprehensive and solid database shall be beneficial for the community to solve the binding and delivery issues of DNA, and to accelerate the development of conjugates for clinical applications.

Third, another potential issue in clinical application of DNA-natural product conjugates is the significantly improved cost when comparing with the small molecule drug due to the DNA conjugation. The cost-effectiveness analysis should be carefully evaluated during the conjugation drug development process. The community could learn from the example of GalNAc-siRNA conjugate which has advanced into multiple preclinical and clinical trials, including three phase III trials initiated by Alnylam. The carefully chosen target receptor and well-designed conjugation chemistry makes the GalNAc-siRNA a successful solution to the RNAi therapeutics for liver hepatocyte diseases (Nair et al., 2017; Thangamani et al., 2021). In the development of DNA-natural product conjugates, the cost-effective nucleic acid manufacturing technologies, such as the use of use of drug-containing phosphoramidite, could be considered to reduce the cost (Zhu et al., 2015). One oligonucleotide strand can also load multiple copies of drug molecules to increase drug loading capacity, thus potentially improving the effectiveness (Beck et al., 2010).

To conclude, the use of DNA as a programmable biorecognition agent to improve natural product drug efficacy is an important and emerging field. Recent progress in the field of molecular biology, biotechnology, and synthetic chemistry may provide better solutions for solving the abovementioned key issues. With more reliable DNA-drug conjugates in hand, the community can meet the critical challenges in the field to develop better pharmacological therapies.
